# Neurologic Adverse Events Associated with Voriconazole Therapy: Report of Two Pediatric Cases

**DOI:** 10.1155/2016/3989070

**Published:** 2016-05-24

**Authors:** Sevliya Öcal Demir, Serkan Atici, Gülşen Akkoç, Nurhayat Yakut, Nilay Baş İkizoğlu, Ela Erdem Eralp, Ahmet Soysal, Mustafa Bakir

**Affiliations:** ^1^Marmara University School of Medicine, Department of Pediatrics, Division of Pediatric Infectious Diseases, 34912 Istanbul, Turkey; ^2^Marmara University School of Medicine, Department of Pediatrics, Division of Pediatric Pulmonology, 34912 Istanbul, Turkey

## Abstract

Although voriconazole, a triazole antifungal, is a safe drug, treatment with this agent is associated with certain adverse events such as hepatic, neurologic, and visual disturbances. The current report presents two cases, one a 9-year-old boy and the other a 17-year-old girl, who experienced neurologic side effects associated with voriconazole therapy. Our aim is to remind readers of the side effects of voriconazole therapy in order to prevent unnecessary investigations especially for psychological and ophthalmologic problems. The first case was a 9-year-old boy with cystic fibrosis and invasive aspergillosis that developed photophobia, altered color sensation, and fearful visual hallucination. The second case was a 17-year-old girl with cystic fibrosis and allergic bronchopulmonary aspergillosis, and she experienced photophobia, fatigue, impaired concentration, and insomnia, when the dose of voriconazole therapy was increased from 12 mg/kg/day to 16 mg/kg/day. The complaints of the two patients disappeared after discontinuation of voriconazole therapy. Our experience in these patients reminded us of the importance of being aware of the neurologic adverse events associated with voriconazole therapy in establishing early diagnosis and initiating prompt treatment. In addition, although serum voriconazole concentration was not measured in the present cases, therapeutic drug monitoring for voriconazole seems to be critically important in preventing neurologic side effects in pediatric patients.

## 1. Introduction

Voriconazole is a broad-spectrum triazole antifungal agent, which is the drug of first choice in the treatment of invasive aspergillosis in adult patients [1]. In European Union, the drug received approval for use in children aged 2 to 11 years in 2005. The increased use of the agent in pediatric population revealed that the compound, particularly its oral formulation, was found to have considerably variable pharmacokinetics in children [2]. Currently, an intravenous dose of 8 mg/kg taken twice daily (9 mg/kg twice daily at day 1) and an oral dose of 9 mg/kg for the oral suspension formulation taken twice daily have been adopted by the European Medicines Agency (EMA) for children aged 2 to <12 years and aged 12 to 14 years weighing <50 kg, and these doses are under further investigation in clinical phase II trials conducted by the manufacturer [3, 4].

Although voriconazole is a safe and often well-tolerated drug, it was found to be associated with some adverse effects such as neurotoxicity, visual disturbances, and dermatologic reactions [5, 6]. Reversible visual disturbances including altered color sensation, photophobia, and blurred vision were reported as the most common side effects accounting for 20 to 30% of adverse reactions in patients [7, 8]. Hallucinations and encephalopathy deserve further investigations for psychological disorders, as these are relatively uncommon adverse reactions associated with voriconazole therapy.

The current report presents two patients with visual and neurological symptoms that occurred during voriconazole therapy. Although the reactions are well-known side effects of voriconazole therapy, we found only a few reports in the literature [9–11], and, thus, the present paper highlighted these known but neglected adverse effects of voriconazole.

## 2. Case  1

A 9-year-old boy was admitted to the hospital due to acute exacerbation of cystic fibrosis. In the first sputum culture, there was growth of* Pseudomonas aeruginosa*, methicillin-sensitive* Staphylococcus aureus*, and methicillin-resistant* Staphylococcus aureus*, and antibacterial therapy was initiated based on the antibiotic susceptibility testing. However, no sufficient clinical improvement was achieved despite use of appropriate doses and duration of antibacterial therapy. The sputum culture performed at day 10 of the hospitalization period showed growth of* Aspergillus fumigatus* for which intravenous voriconazole therapy was initiated at a loading dose of 9 mg/kg twice daily at day 1 followed by the maintenance dose of 8 mg/kg twice daily. In the first day of the treatment, the patient developed photophobia and he continuously winked his eyes approximately for 10 minutes during each voriconazole infusion. At day 2, the patient winked his eyes approximately for 15 minutes during voriconazole infusion, and the patient additionally suffered from seeing ant colonies, and he reported that his mother had dressed with clothes with pink and red flowers, although the mother was actually dressed in black. The patient kept his eyes closed to avoid these hallucinations. These symptoms were considered as drug reactions, as the patient did not have a previous history of psychological problems or did not previously exhibit similar symptoms. Therefore, the treatment was switched to caspofungin and all symptoms disappeared. There were no further complaints one month after discontinuation of voriconazole therapy.

## 3. Case  2

A 17-year-old female patient with cystic fibrosis and allergic bronchopulmonary aspergillosis (ABPA) was admitted to our outpatient clinics due to increased sputum production and cough for the last fifteen days. Pulmonary auscultation revealed wheezing, while repeated measurement of total serum IgE was found to be elevated from 536 IU/mL to 1508 IU/mL and sputum cultures showed no growth. Based on these findings, we considered exacerbation of ABPA, and the dose of voriconazole therapy was increased from 200 mg twice daily to a maximum daily dose of 600 mg in order to reduce antigenic burden by combating fungal infection of the airway. However, the patient suffered from fatigue and impaired concentration a week after, and she developed photophobia and anxiety, which persisted approximately for four hours following oral dose of voriconazole. These symptoms were considered to be suggestive of a drug reaction. Voriconazole therapy was discontinued and all symptoms disappeared.

## 4. Discussion

Voriconazole is the drug of first choice in the treatment of invasive aspergillosis. Voriconazole trough concentrations >1 *µ*g/mL were reported to be associated with good clinical response to therapy and improved survival. However, due to nonlinear pharmacokinetics of voriconazole in children, achieving this concentration in this group may be challenging. Data suggest that children <12 years have approximately a 50% reduction in bioavailability for voriconazole compared to adults, which suggests that higher doses are required for children to provide the same effects [12, 13]. Currently, the dose of voriconazole used in children ranges from 3.4 to 23 mg/kg/d [14].

A strong correlation has been suggested between higher voriconazole trough concentrations and neurologic side effects, although this still remains controversial [11, 15]. Zonios et al. [9] suggested that there might be an increased risk of hallucination in patients with levels greater than the mean voriconazole level. Likewise, Imhof et al. [11] also reported a significant association between elevated serum voriconazole levels (sVL) and occurrence of neurological symptoms within 3 and 22 days (median 7 days) after the initiation of voriconazole therapy or dosage adjustment [11]. Our first case experienced visual disturbances and hallucination during voriconazole infusion at a loading dose of 9 mg/kg. The incidence of hallucinations associated with voriconazole therapy was reported to range from 4.3% to 16.6% in various studies [9, 16], whereas reversible visual disturbances occurred in 20 to 30% of patients. On the other hand, we must be aware of the fact that differentiation of visual hallucination from visual disturbances can be challenging. Beside hallucination and visual impairment, Imhof et al. reported nonspecific signs of encephalopathy during voriconazole treatment such as fatigue, impaired concentration, loss of memory, insomnia, anxiety, irritability, and dysarthria [11]. Except dysarthria, all of these signs were reported by the second case after voriconazole dose has been increased to a maximum daily dose of 600 mg/d. Due to the fact that voriconazole levels (VL) are not measured at our institution, VL of our cases were not available. Hepatotoxicity, renal toxicity, arrhythmia, or rash was not observed during the follow-up period. In a large cohort of immunocompromised children, Pieper et al. [17] reported their eight-year experience on the adverse effects of voriconazole therapy; elevated hepatic transaminases, serum bilirubin, and alkaline phosphatase levels, skin eruptions, and neurological adverse effects were observed in 53.5, 23.6, 10.9, 5.6, and 4.8% of the courses of voriconazole therapy, respectively. Photophobia was noted in eight cases, visual hallucination in one, insomnia in one, vertigo in one, and lack of concentration in one [17].

No specific treatment was required for neurological symptoms associated with voriconazole therapy, and discontinuation of drug was sufficient to control symptoms in the present cases as was reported in a prospective study by Zonios et al. [9]. In addition, switching therapy from intravenous formulation to oral formulation could be sometimes sufficient or mild symptoms can be observed at the beginning of the voriconazole therapy, which later disappear despite continuation of the therapy [9]. Therefore, making an attempt to initiate an oral formulation of voriconazole before discontinuing intravenous formulation can be considered in some critically ill patients. In our first case that received intravenous voriconazole therapy, due to the deterioration in medical condition of the patient and concerns of their parents, we switched to another antifungal agent, caspofungin, instead of proceeding with oral voriconazole therapy.

In conclusion, neurologic adverse effects associated with voriconazole therapy should be kept in mind in order to early recognize and initiate prompt treatment for these conditions and to avoid unnecessary investigations. Although serum voriconazole levels were not evaluated in the present study, monitoring of serum voriconazole levels might increase efficiency and safety of therapy particularly in pediatric patients.
